# Label-free multiphoton microscopy enables histopathological assessment of colorectal liver metastases and supports automated classification of neoplastic tissue

**DOI:** 10.1038/s41598-023-31401-5

**Published:** 2023-03-15

**Authors:** Roberta Galli, Tiziana Siciliano, Daniela Aust, Sandra Korn, Katrin Kirsche, Gustavo B. Baretton, Jürgen Weitz, Edmund Koch, Carina Riediger

**Affiliations:** 1 Department of Medical Physics and Biomedical Engineering, Faculty of Medicine Carl Gustav Carus, Technische Universität Dresden, Fetscherstr. 74, 01307 Dresden, Germany; 2 Center for Regenerative Therapies (CRTD), Technische Universität Dresden, Fetscherstr. 105, 01307 Dresden, Germany; 3 Institute of Pathology, University Hospital Carl Gustav Carus, Medical Faculty, Technische Universität Dresden, Fetscherstr. 74, 01307 Dresden, Germany; 4 National Center for Tumor Diseases (NCT/UCC), Partner Site Dresden: German Cancer Research Center (DKFZ), Im Neuenheimer Feld 280, 69120 Heidelberg, Germany; 5 Department of Visceral, Thoracic and Vascular Surgery, University Hospital Carl Gustav Carus, Technische Universität Dresden, Fetscherstr. 74, 01307 Dresden, Germany; 6 Neurosurgery, University Hospital Carl Gustav Carus, Technische Universität Dresden, Fetscherstr. 74, 01307 Dresden, Germany; 7 Clinical Sensoring and Monitoring, Department of Anesthesiology and Intensive Care Medicine, Faculty of Medicine Carl Gustav Carus, Technische Universität Dresden, Fetscherstr. 74, 01307 Dresden, Germany

**Keywords:** Biophysics, Cancer, Oncology, Optics and photonics

## Abstract

As the state of resection margins is an important prognostic factor after extirpation of colorectal liver metastases, surgeons aim to obtain negative margins, sometimes elaborated by resections of the positive resection plane after intraoperative frozen sections. However, this is time consuming and results sometimes remain unclear during surgery. Label-free multimodal multiphoton microscopy (MPM) is an optical technique that retrieves morpho-chemical information avoiding all staining and that can potentially be performed in real-time. Here, we investigated colorectal liver metastases and hepatic tissue using a combination of three endogenous nonlinear signals, namely: coherent anti-Stokes Raman scattering (CARS) to visualize lipids, two-photon excited fluorescence (TPEF) to visualize cellular patterns, and second harmonic generation (SHG) to visualize collagen fibers. We acquired and analyzed over forty thousand MPM images of metastatic and normal liver tissue of 106 patients. The morphological information with biochemical specificity produced by MPM allowed discriminating normal liver from metastatic tissue and discerning the tumor borders on cryosections as well as formalin-fixed bulk tissue. Furthermore, automated tissue type classification with a correct rate close to 95% was possible using a simple approach based on discriminant analysis of texture parameters. Therefore, MPM has the potential to increase the precision of resection margins in hepatic surgery of metastases without prolonging surgical intervention.

## Introduction

Colorectal liver metastases (CRLM) are the main indication for hepatectomy worldwide. However, only 20–30% of all liver metastases are potentially curative resectable^1^. The rate of resectable CRLM has been raised over the last decades by converting chemotherapy^2,3^. Incomplete resections with macroscopic residual tumor (R2) should be avoided due to fatal prognosis. Even though controversially discussed in the era of chemotherapy and target treatments, resection margins are a relevant prognostic factor for disease-free and overall survival^4–9^. Incomplete resections with microscopic residual tumor (R1) are mostly associated with a poorer prognosis and therefore the surgery aims to achieve tumor-free resection margins (R0). The gold standard for the evaluation of resection margins is histopathological analysis. The postoperative evaluation of resection margins is performed using hematoxylin/eosin (HE) staining as well as immunohistochemistry of formalin-fixed and paraffin-embedded sections. On the other hand, fast evaluation for intraoperative information is based on fresh frozen sections of unfixed tissue using limited staining methods that often lead to unclear or incoherent results. In addition, obtaining information about resection margins is time consuming owing to specimen transportation and analyses, which result in prolonged operation times of at least 20–30 min. However, intraoperative histopathological analyses are important as intraoperative knowledge of R1 resection margin can mostly be corrected by resection of the former resection plane. To overcome waiting time and unclear information, new methods to evaluate the resection planes are needed and should be investigated. A promising approach is the use of label-free multiphoton microscopy for intraoperative tissue imaging.

Multimodal multiphoton microscopy (MPM) enables the analysis of tissue structures down to subcellular resolution based on the morpho-chemical information retrieved from optical signals generated by endogenous tissue constituents upon irradiation with short-pulsed lasers. In medicine, the technique is gaining increasing attention for fast pathology without tissue preparation and intravital microscopy. In addition, label-free MPM can combine the simultaneous acquisition of different nonlinear optical signals, which has proven effective for tumor delineation^10,11^.

Especially useful for this purpose is the combination of coherent anti-Stokes Raman scattering (CARS), two-photon excited fluorescence (TPEF) and second harmonic generation (SHG). For instance, CARS allows imaging of lipid-rich tissue structures (e.g., cell membranes and lipid droplets), SHG of fibrillary collagen (e.g., in fibrotic tissue and blood vessels) and TPEF of other endogenous intra- and extra-cellular fluorophores (elastin, NAD(P)H, FAD, lipofuscin). This approach was exploited especially as a diagnostic and prognostic alternative method for the diseased central nervous tissue^12,13^, also using unprocessed human biopsies^14^. The identification of the tumorous area in other organs, such as the kidney, was likewise demonstrated based on a combination of morphological and biochemical features retrieved in a label-free manner^15^. Furthermore, it was shown that automated image analysis—such as morphometric or texture analysis—of label-free multiphoton images obtained by the combination of CARS, TPEF and SHG can provide an objective tool for tumor recognition besides the visual inspection by a trained pathologist^16,17^.

Photochemical alterations during MPM are minimized by using near-infrared laser excitation and lack of tissue damage was proven in vivo in animal models using normal working conditions; moreover, any photochemical alteration eventually induced during MPM imaging can be promptly recognized on a variety of tissue types, including the liver^18^. On the other side, the feasibility of endoscopic label-free multimodal multiphoton microscopy, including CARS, has been demonstrated both based on rigid endoscope^19,20^ as well as optical fibers^21,22^. Commercial endoscopic systems are not available yet, but—in principle—both techniques have a potential for further miniaturization, as required by medical products foreseen for intraoperative use.

Despite the great potential, label-free MPM integrating CARS is still largely unexplored for hepatic tumors, whose treatment may also gain great benefit from precise intraoperative detection of tumor boundaries. Multimodal imaging integrating CARS was only used to analyze liver steatosis and fibrosis, providing the ability to visualize hepatic fats, collagen fibrils, and hepatocyte morphology^23–26^. Interestingly, it was shown that computer-assisted analysis of liver lipid levels based on the CARS signal intensity is consistent with the triglyceride measurement using a standard biochemical assay^27^. Further studies aimed to indirectly evaluate steatosis simply based on the lack of nonlinear signals (e.g. TPEF) in the holes left after section dewaxing, eventually in combination with SHG to detect fibrosis^28,29^. The latter was extensively addressed by visualization of the collagen fibers using SHG microscopy, eventually complemented by TPEF to retrieve cellular structures^30–37^. Automated evaluation of liver fibrosis using SHG and TPEF microscopy was compared with Ishak fibrosis scores and other currently used quantitative methods for determining liver fibrosis, showing that morphological parameters retrieved with label-free MPM faithfully recapitulate the standard fibrosis scores and thus can be used for quantitative staging of liver fibrosis^38^.

Only few studies aimed to differentiate cancer from liver tissue using label-free MPM. The combination of TPEF and SHG was used in a preclinical study to diagnose liver cancer and differentiate benign and malignant liver lesions such as hepatocellular carcinoma (HCC) and metastatic lesions, including CRLM^39^. In rat models, it was similarly shown that TPEF and SHG illustrate the tissue architecture and subcellular morphology enabling the diagnose of liver cancer and lung metastasis^40^. The automated classification of TPEF and SHG images based on deep learning was applied for recognizing and grading HCC and provided a classification accuracy above 90%^41^. To our best knowledge, studies on liver cancers that also exploited CARS are not available so far.

Recently, it was shown that an approach based on label-free MPM of liver tissue integrating CARS, TPEF and SHG followed by texture feature extraction and linear discriminant analysis is able to recognize early septic liver injury in a murine model. For this purpose, CARS and TPEF showed excellent discrimination between sections of septic mice and sham-treated mice, in contrast to SHG^42^. On the other side, a similar approach based on texture analysis of CARS and TPEF images followed by discriminant analysis provided automatic recognition of brain tumors with accuracy up to 96%^43^.

Here, we investigated tumor and liver tissue samples of patients that underwent surgery for treatment of colorectal liver metastases by using label-free multimodal imaging with CARS, TPEF and SHG. Images were visually analyzed to retrieve main morphological differences among tumor and liver tissue samples within the large inter-patient variability. The classification of image texture parameters by linear discriminant analysis was exploited for automated tissue type recognition. Whereas high-resolution images were used for visual inspection down to subcellular details, low-resolution images acquired at a frame rate of about 3 fps were used for automated classification in order to simulate a fast intraoperative application for identification of tumor tissue at the resection planes. This study aims to generate the basic knowledge necessary for the translation in the clinics, which should be based on fast imaging of freshly resected tissue samples with multiphoton microscopes or in situ tissue imaging using endoscopic devices.

## Results

### Label-free multiphoton microscopy enables histopathological assessment of CRLM on cryosections and bulk tissue samples

**Figure 1:**
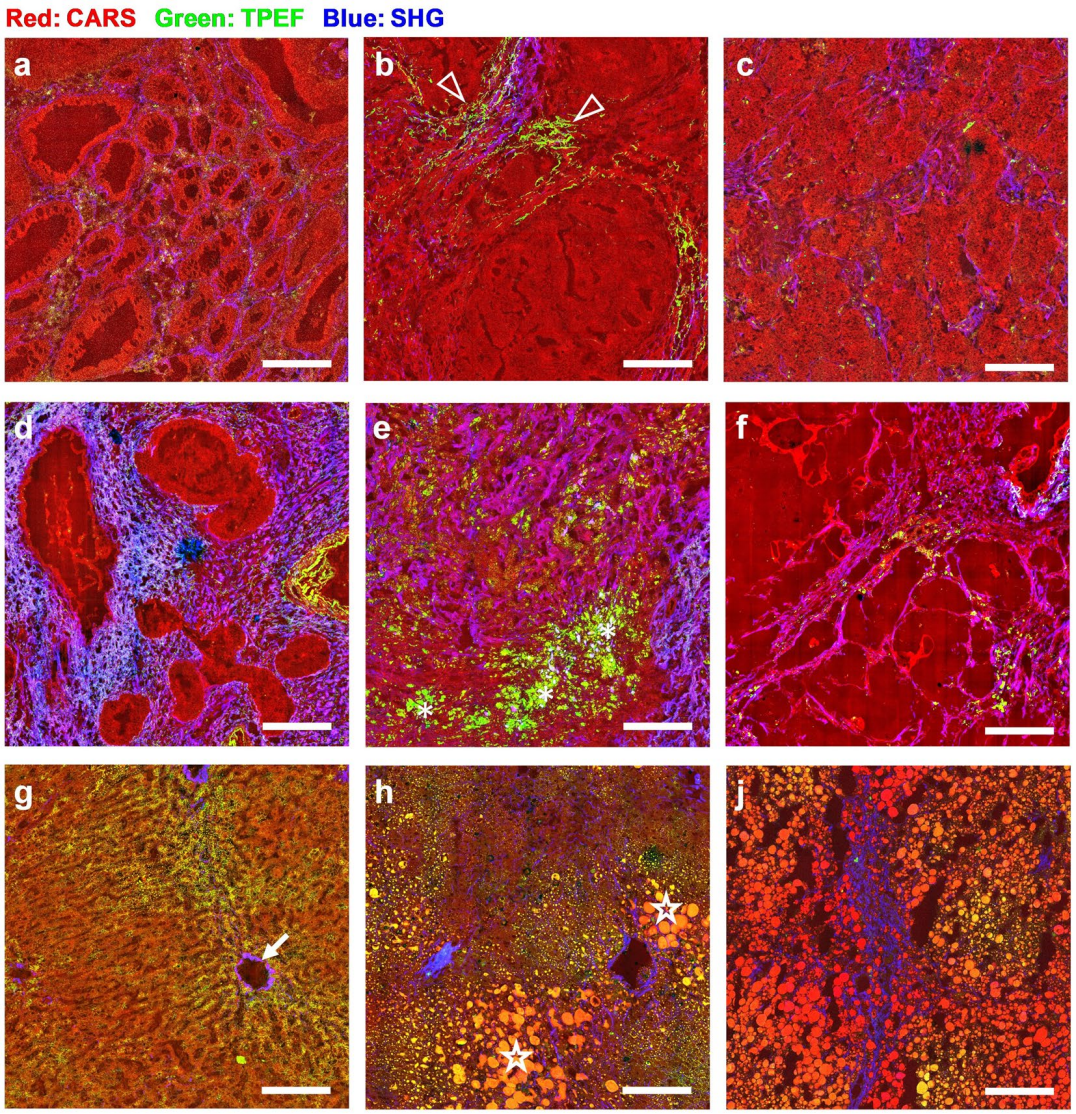
Macro-morphology of CRLM tissue vs. non-metastatic liver tissue shown by MPM imaging of cryosections. (**a**)–(**f**): metastatic tissue; (**g**)–(**j**): non-metastatic liver tissue. Scale bar: 200 µm. HE staining of the same sections is shown in Supplementary Fig. S1.

**Figure 2:**
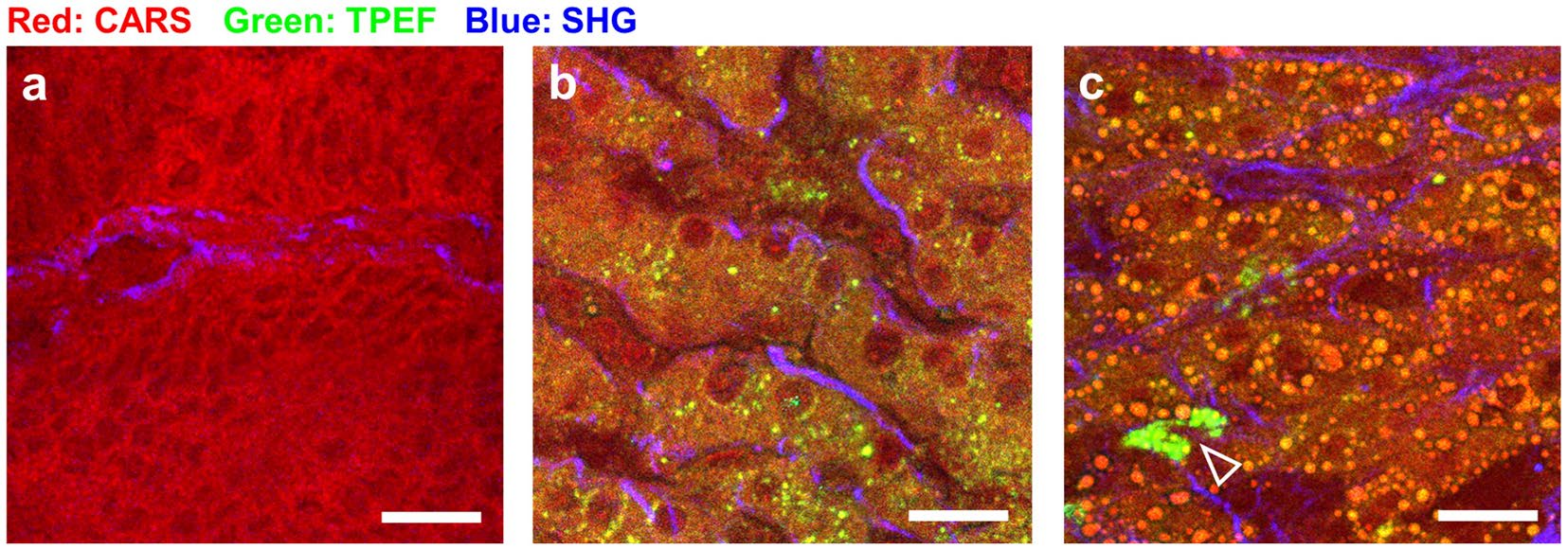
Micro-morphology of CRLM tissue vs. non-metastatic tissue as shown by MPM imaging of cryosections. (**a**): Metastatic tissue; (**b**, **c**): non-neoplastic liver tissue. The arrowhead indicates autofluorescent immune cells. Scale bar: 20 µm. HE staining of the same sections is shown in Supplementary Fig. S2.

The identification of tumor and normal tissue characteristics was performed by visual inspection of high-resolution MPM images of cryosections. The tissue macro- and micro-morphology can be clearly recognized (Figs. 1 and 2). Generally, both cellular morphology and lipid droplets are displayed by CARS (red channel); collagen and elastin fibers are visualized by SHG (blue channel) and TPEF (green channel), respectively. Moreover, TPEF is emitted by several intracellular fluorophores and can be seen in the cytoplasm of different cell types. All structures shown by MPM were matched and verified using HE stained sections (the very same section stained after MPM, or a consecutive one), shown in Supplementary Figs. S1 and S2.

The morphology of metastatic tissue displayed a rather high degree of variability, which is illustrated in Fig. 1a–f. About 80% of the CRLM samples show a glandular morphology resembling the primary colorectal adenocarcinoma^44^. These samples display glands lined by columnar epithelial cells, often with a well-defined lumen (Fig. 1a), or show cells organized in sheets and nests (Fig. 1b). Fewer samples (less than 10%) show a rather homogenous tumor structure without a specific organization of tumor cells (Fig. 1c). As also known for the primary cancer, the metastatic tissue is additionally characterized by the presence of variable amounts of collagenous stroma among the malignant cells^45^, as shown in the example in Fig. 1a with thin strands of stroma between the glandular structures, and Fig. 1d with very thick fibrous regions surrounding the glandular structures. Moreover, elastin was detected by TPEF in the extracellular matrix of some tumors, as visible in the samples of Fig. 1b (arrowheads). In the remaining 10% of samples, extensive fibrosis and necrosis were detected, mostly lacking vital tumor tissue. As shown by Fig. 1e, tissue morphology of such samples is dominated by a dense mesh of collagen fibers intermingled by necrotic regions of variable extension with accumulation of fluorescent cells and tissue debris (asterisks), so that a clear tumor morphology as described above for Fig. 1a–c was not visible. In general, necrosis and fibrosis are the consequence of preoperative chemotherapy^46^ and were detected with variable extensions in about half of the tumor samples. In three samples, a very loose tumor structure was visible, with large pools separated by thin strands of tumor or fibrotic tissue and containing floating cells (Fig. 1f). This morphology indicates a mucinous adenocarcinoma type^47^ and the HE staining shows indeed mucin pools (Supplementary Fig. S1f).

The non-neoplastic samples included normal liver tissue without other visible pathologies, as shown in Fig. 1g, as well as altered liver tissue with different degrees of steatosis and fibrosis, mainly as a consequence of chemotherapy-associated liver injury^48^. In the normal liver parenchyma, hepatocytes are arranged in sort of cords one or two cells thick, which are organized around a terminal hepatic venule (central vein, indicated by an arrow in Fig. 1g). Moderate steatosis (Fig. 1h) is characterized by regions with lipid droplets displayed very well by CARS (stars in Fig. 1h). In the severe steatosis, accumulation of lipid droplets is observed in large tissue regions as shown in Fig. 1j; here, also fibrosis was detected by SHG.

In all acquired images, the metastatic tissue was discernible from non-neoplastic tissue based on the evaluation of the above-described morphological characteristics. Also, at higher magnification, the metastatic tissue (Fig. 2a) differs in cellular density and morphology from the normal liver tissue (Fig. 2b) also when cytoplasmic lipid droplets are present (Fig. 2c).

**Figure 3:**
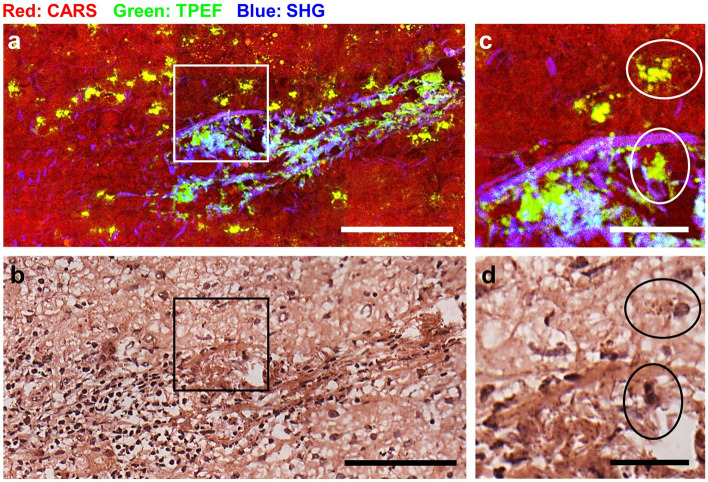
Inflammatory cells visualized by endogenous TPEF in metastatic tissue. (**a**): MPM image of tissue cryosection; (**b**): HE staining of the same tissue cryosection. (**c**): Magnification of the area in the white box in (**a**); (**c**): magnification of the area in the black box in (**b**). Fluorescent cells with recognizable nuclei in the HE staining are indicated. Scale bar in (**a**, **b**): 100 µm; scale bar in (**c**, **d**): 25 µm.

Additionally, TPEF offers the possibility to address a subset of inflammation cells. It was shown that activated immune cells display an intense endogenous TPEF signal of NAD(P)H, which is expressed during the oxidative burst^49–51^. In the liver, highly fluorescent cells were detected in many samples of both tumor and normal tissue localized near blood vessels, as well as scattered within the tissue (Fig. 3). Generally, they were observed more often in tumor samples. Based on the intense autofluorescence, also the cells indicated by the arrowhead in Fig. 2c can then be recognized as immune cells.

**Figure 4:**
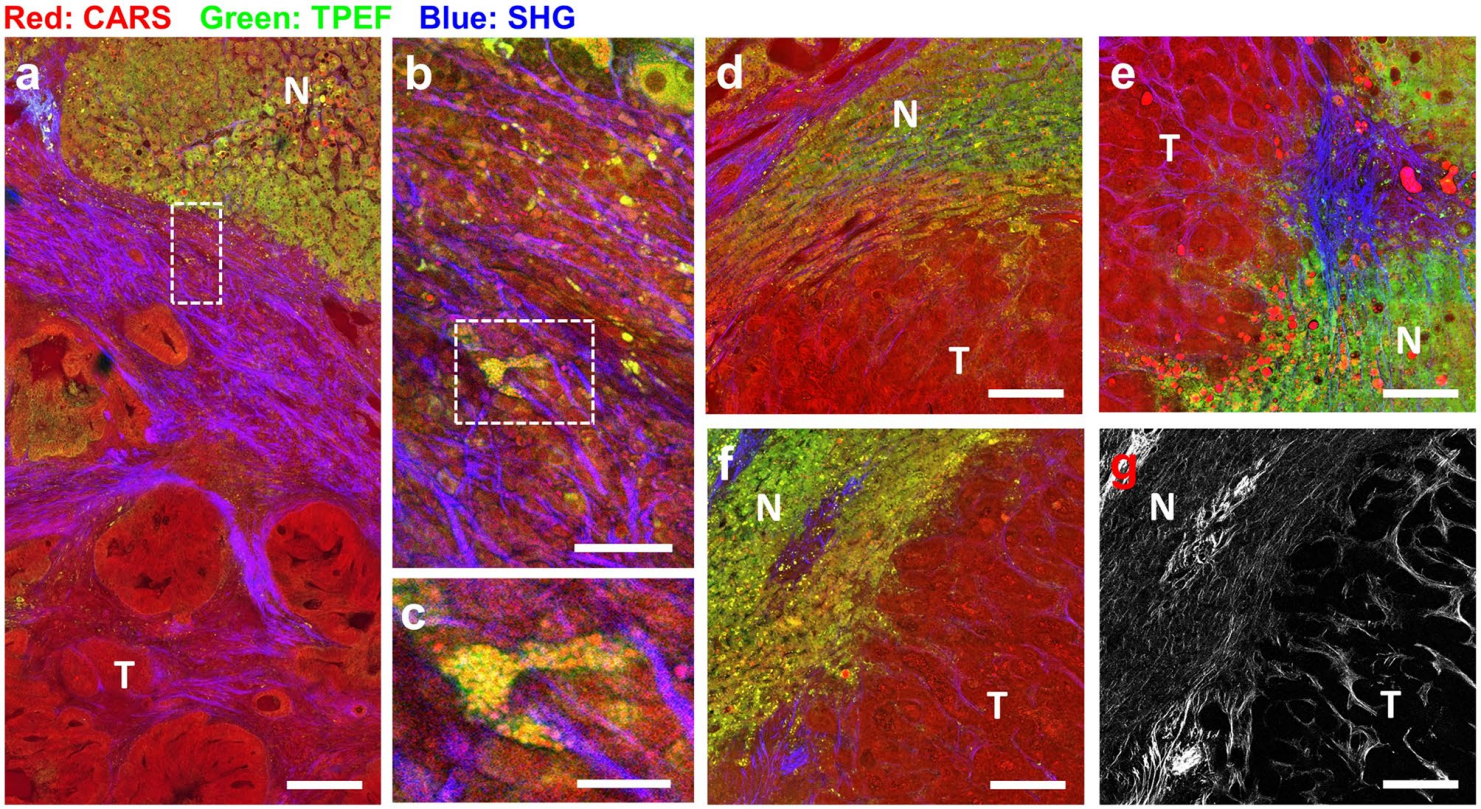
Histological growth patterns of liver metastases shown by MPM imaging of formalin-fixed tissue blocks. (**a**): Desmoplastic HGP; (**b**): magnification of the border region in the box in (**a**); (**c**): Magnification of the region in the box in (**b**), showing lipid-laden foam cells; (**d**): pushing HGP; (**e**): replacement HGP; (**f**): intermediate HGP between pushing and replacing; (**g**) SHG channel only showing the different morphology of collagen fibers in normal hepatic parenchyma (reticulin) vs. metastatic tissue. Scale bar in (**a**), (**d**–**g**): 200 µm; scale bar in (**b**): 50 µm; scale bar in (**c**): 20 µm. N: normal liver tissue, T: tumor. HE staining of the same sections is shown in Supplementary Fig. S3.

The possibility of distinguishing prognostic tumor growth patterns^52^ was investigated using formalin-fixed bulk tissue samples specifically resected by the surgeon at the tumor margins. All three main forms of histological growth patterns (HGP)^53^ of CRLM were found in the analyzed samples. Examples are shown in Fig. 4 (see Supplementary Fig. S3 for reference HE staining). In the desmoplastic HGP (Fig. 4a), the rim of collagenous stroma that separates the metastatic tissue from the surrounding liver parenchyma is visualized by SHG. Typically, the desmoplastic rim presents a more dense part toward the tumor and a less dense part toward the liver tissue harboring a high number of immune cells. Some of these could be identified by TPEF (see detail in Fig. 4b); fluorescent foam cells laden with lipid droplets are also present (Fig. 4c). In the pushing HGP (Fig. 4d), the liver plates are pushed aside and compressed by the tumor growing front, running parallel with the metastases’ border. No desmoplastic stroma was detected at the interface to the tumor. In the replacement HGP (Fig. 4e), tumor cells co-opt the parenchyma structure replacing the hepatocytes with no detectable inflammatory cells and fibrosis. The reticulin network of the liver parenchyma is visualized by SHG. It is not conserved within the metastases, which show altered collagen structures, typically separating the nests of tumor cells (Fig. 4f,g).

### Label-free multiphoton microscopy supports automated classification of metastatic tumor vs. normal liver tissue

For automated classification, the low-resolution images of cryosections were used. Altogether, 47,167 single FoV images with a resolution of 1 µm/pixel were acquired on cryosections of CRLM and normal liver tissue of 76 patients using an automated tiling procedure. After discarding all images containing tissue borders, large holes or other artefacts (e.g. dirt, air bubbles, artefacts due to the saturation of detectors; see examples in Supplementary Fig. S4), 40,392 single FoV images were passed to texture analysis and used for classification.

Thirty-two patients with both matched tumor and normal tissue were randomly selected and assigned to the test set. All other patients (27 patients with both tumor and normal tissue, 9 patients with tumor tissue only and 6 patients with normal tissue only) were used as training set. The test set included 18,868 images (8829 images of normal tissue samples and 10,039 images of tumor tissue samples). For each patient in the test set, a median of 310 images were available (min: 40, max: 734). The training set included 21,524 images (9890 images of normal tissue samples and 11,634 images of tumor samples). For each patient in the training set, a median of 267 images were available (min: 83, max: 700).

From each signal channel (i.e. CARS, TPEF and SHG), five first-order and twelve second-order texture parameters were calculated and afterwards used for image classification by discriminant analysis. The correct reclassification rate with leave-one-sample-out cross-validation (i.e. classify all images of one sample using all other images as training set) was used as a benchmark to select the classification model (linear or quadratic) and the texture parameters to be used. The best classification performances were obtained using all 17 texture parameters retrieved from CARS and TPEF channels but only 8 texture parameters from the SHG channel (the 5 first order parameters plus contrast for three distances) and linear discriminant analysis. With this approach, the average correct rate of image classification was 95.4% (specificity: 98.2%, sensitivity: 93.8%; correct classification rate for normal tissue: 98.4%, correct classification rate for tumor tissue: 92.3%).

**Figure 5:**
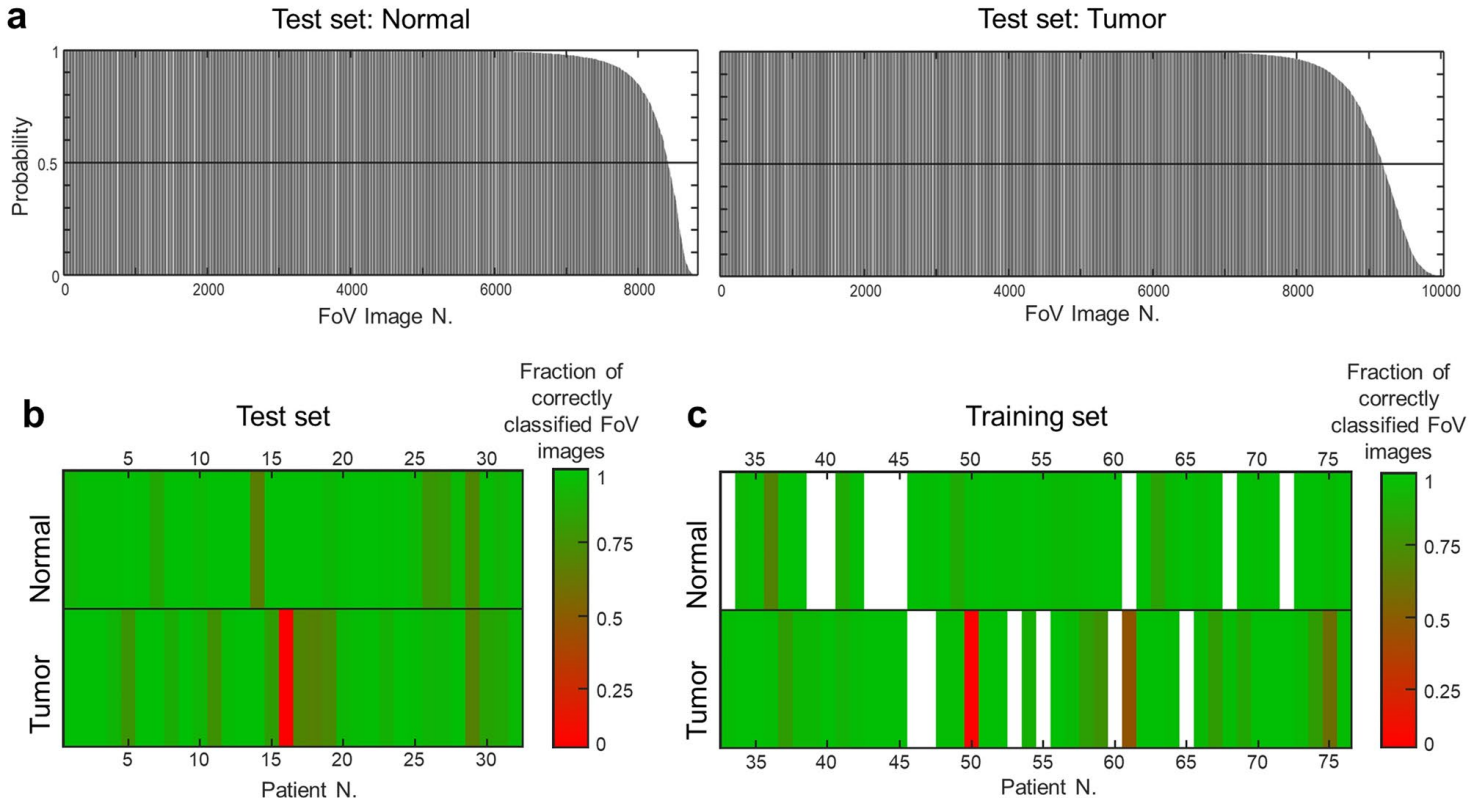
Classification of tumor vs. normal tissue. (**a**) Posterior probability of group attribution for each single FoV images of normal and tumor tissue in the test set. (**b**) Graphical representation of the fraction of correctly classified images for each patient in the test set. (**c**) Graphical representation of the fraction of correctly reclassified images for each patient in the training set.

The classification model was then applied to the images of the independent test set for validation. An overall correct rate of 93.4% was obtained for images (specificity: 95.6%, sensitivity: 90.9%; correct classification rate for normal tissue: 95.4%, correct classification rate for tumor tissue: 91.6%). Moreover, about 70% of images were correctly classified with a posterior probability close to 1 (Fig. 5a); only a small part of images had a posterior probability close to 0.5, which is interpreted as inconclusive attribution.

The Supplementary Table S1 reports the fraction of single images that were correctly classified for each patient of test and training set. Figure 5b,c shows this information graphically. Only for one tumor sample in the test set (Patient 16) and one in the training set (Patient 50), the large majority of images were false classified. One tumor sample in the training set (Patient 61) is above but very close to 0.5. The samples of normal tissue were all correctly classified.

All samples with less than 85% of correctly classified images were analyzed in order to identify possible reasons for misclassification.

For samples with a high fraction of misclassified images, the reason was evident already from the visual inspection of images. The tumor sample of patient 16 in the test set is mainly necrotic with no viable tumor tissue. Furthermore, due to the low quality of the tissue because of necrosis, just a few images were left for analysis after discarding the ones with holes and artefacts. The tumor sample of patient 50 in the training set does contain in fact only normal tissue, as shown by the MPM image and as confirmed by analysis of the histological staining by a pathologist. The tumor sample of Patient 61 (also in the training set) shows large accumulations of fats, fibrosis and necrosis. These tumor samples are shown in Supplementary Fig. S5a–c. The normal samples of patients 14 and 29 of the test set were also partly misclassified (about 30% of single images attributed to tumor). Both samples display severe steatosis and fibrosis, with large tissue regions lacking almost completely normal hepatocytes. A detail of the normal sample of Patient 14 is shown in Fig. 1j; the normal sample of Patient 29 is shown in Supplementary Fig. S5d.

**Figure 6:**
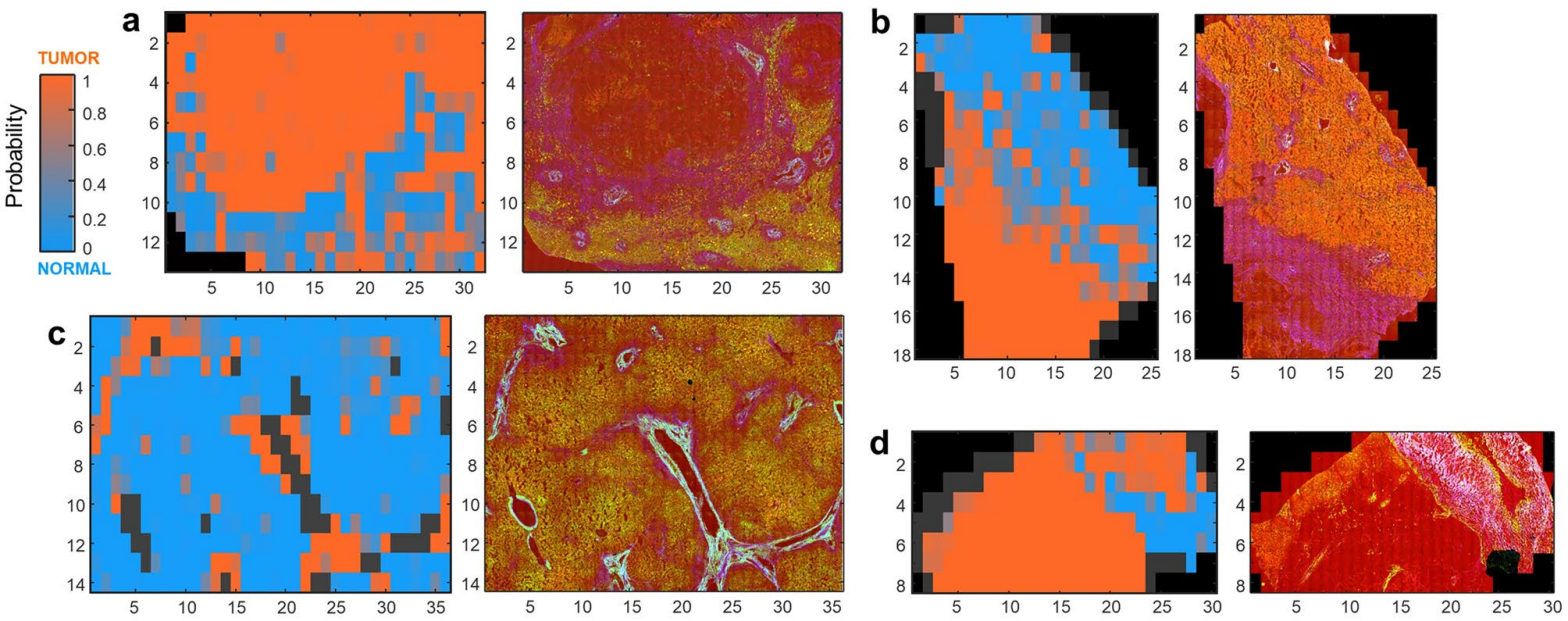
Analysis of misclassified images. The posterior probability of classification of each single FoV image was color-coded as follows: 100% probability tumor: orange; 50% probability tumor: gray; 0% probability tumor: light blue. The positions of single images eliminated before classification (sample borders and tissue holes) are identified by dark gray. For each sample, the map of posterior probability with the tiled MPM image is shown. (**a**) Tumor sample of patient 19; (**b**) tumor sample of patient 29; (**c**) normal sample of patient 27; (**d**) tumor sample of patient 5. Axes’ scale indicates the number of images.

For all other samples that were partly misclassified, a deeper analysis was required to identify the misclassification causes of each single FoV image. For this purpose, the posterior probability of classification was plotted for each sample in order to create maps enabling to identify the misclassified FoV images in the overall tiled multiphoton images and matching them with the corresponding HE staining (see Fig. 6).

Most samples with a rather large number of misclassified single FoV images were tumor samples. For these samples, it was possible to associate the major part of the false classified image with regions of normal tissue that were all recognized by a pathologist based on retrospective analysis of HE staining of the very same or consecutive section. This is the case of samples of Patients 11, 18, 19 and 29 of the test set, as well as 50, 59 and 75 of the training set. Two examples (Patients 19 and 29 of the test set) are shown in Fig. 6a,b.

Another cause underlining misclassification of images of both normal and tumor samples is the massive presence of connective tissue within the walls of large blood vessels. Two examples of a normal tissue sample (Patient 27 of the test set) and a tumor sample (Patient 5 of the test set) are shown in Fig. 6c,d, respectively. Regions of connective tissue that were wrongly classified by the algorithm were also found in the tumor sample of Patient 17 and in the normal samples of Patient 26, both in the test set. The fibrous tissue is attributed by the algorithm to normal or tumor, depending on the sample. The exact reason for class attribution of fibrous tissue remained unclear.

Only for the normal sample of Patient 36 in the training set the reason for misclassification of a part of images could not be fully clarified, although a rather severe fibrosis was detected.

The classification algorithm developed on the training set of cryosections was tested to classify images acquired on formalin-fixed bulk samples. As the samples contain the border between tumor and normal tissue, no direct reference was available for attributing single FoV images to normal or tumor tissue. Therefore, a correct classification rate for single images could not be calculated. However, it was clear by analyzing the results for each single sample that the classification did not work properly. This was explained by the alteration of tissue autofluorescence after formalin fixation, being fixed tissue is far more fluorescent compared with frozen tissue. Interestingly, the same classification approach developed using only the CARS texture parameters of the training set of cryosection was able to identify regions of tumor and normal tissue within most samples. The posterior probability was not that high as for classification using texture parameters of all signals, and, similarly to cryosections, the classification of regions with an accumulation of collagen as well as the necrotic area within the tumor was partly misclassified. Examples are shown in Supplementary Figs. S6 and S7.

## Discussion

Complete resection of CRLM is of high importance regarding patients’ oncological prognosis. The width of the resection margins is discussed controversially, especially concerning neoadjuvant chemotherapy, size of the primary tumor and BRAF/KRAS mutation^4–9^. Although controversial reports have been published analyzing the impact of microscopically incomplete resection on disease-free (DFS) and overall survival (OS), most studies showed significant reduced DFS and OS in patients with R1 resections. In the 1980s and 1990s, resection margins of at least 1 cm were recommended in the hepatectomy for CRLM^54,55^. Later, other studies showed the importance of negative resections margins, but independently of the width of the margin^56,57^. Noteworthy, the definition of R1 and R0 resection varies between the different studies (8). Some authors define R0 with tumor-free tissue margin > 1 mm, while others define 0 mm^58–60^. This leads to different results regarding R1 resection rates in the literature. In recent reports, tumor-negative resection margin of at least 1 mm is associated with improved DFS and OS in patients with isolated CRLM^61,62^. However, up to 30% of positive resection margins are reported for CRLM^63^.

Immediate intraoperative information about resection margins is essential, as re-resection of the former resection plane can easily be performed during surgery, converting R1 into R0 margins. The gold standard for intraoperative information about resection margins is histopathology. Histopathological analyses are performed by staining fresh frozen sections or using unfixed fresh tissue. Due to the transportation and staining process, results can be obtained after 15 min in the best cases. In most cases, the time interval from taking tissue samples to obtaining information becomes significantly long, so that a waiting time of 30–40 min is not uncommon. Consequently, there is an urgent need for new techniques to obtain immediate information about the resection margins.

Achterberg et al. reported the use of ICG-fluorescence to obtain real-time surgical assessment in minimally invasive resection of CRLM. After good preliminary results, the reported pilot trial is currently conducted as a national multicenter trial^64^. However, application of ICG is needed 24 h before surgery for the use of ICG fluorescence. In times of fast track and short hospital stay, this might lead to logistic problems in daily practice. Moreover, the specificity of ICG for different states of CRLM (without pretreatment, necrotic, etc.) is comparably low.

For the use of label-free multiphoton microscopy, no drug or substance application is necessary, so that real-time information can be retrieved. Histopathological analysis of frozen sections for intraoperative evaluation of resection margins could be replaced by label-free MPM of unprocessed small tissue biopsies taken from the resection planes.

Label-free multiphoton microscopy combining CARS, TPEF and SHG enables to discern metastatic structures from normal liver parenchyma by visual inspection of images and is highly specific to differentiate CRLM from liver tissue. When coupled to artificial intelligence methods for automated image classification, it might support the surgeon in the optimization of the resection planes substituting intraoperative frozen sections. Acquisition of high-resolution images with long acquisition time is not needed for automated classification. As low-resolution images suited for classification can be registered with a frame rate of about 3 fps, the acquisition of enough images for a sound evaluation of a small tissue sample may require a couple of minutes. The classification exploits computationally simple algorithms. Therefore, after building the classification algorithm on a test set, the extraction of texture parameters from each single image needs just some millisecond, similar to image classification. If a suited multiphoton microscope is available near the OR, the surgeon may use the classification results to optimize the resection planes almost in real-time.

Our classification algorithm was able to differentiate metastatic from liver tissue whenever viable tumor tissue was available in the analyzed samples. The morphological heterogeneity of both metastatic and liver tissue (as shown in Figs. 1 and 2) did not prevent the algorithm to work properly. Furthermore, the attribution of each single field-of-view image to the class “tumor” or “normal” was clear-cut, as just a small fraction of images had a posterior probability of around 0.5. The reason for misclassification became clear in most cases by retrospectively analysis of misclassified MPM images in comparison with HE staining. Regions of tumor samples were recognized as normal tissue because they were indeed made of normal liver tissue: this demonstrates the robustness of the classification. The remaining tumor images that were wrongly classified mainly contained large blood vessels or highly fibrotic tissue, as well as necrotic areas mostly detected in patients that received preoperative chemotherapy.

In this study, the algorithm was not able to assign correctly the fibrotic tissue to the classes “tumor” or “normal”, probably because it was equally present both in normal and in tumor samples. Misclassification of normal tissue could be explained by the massive presence of fibrotic tissue as well as severe steatosis. However, both constitute a problem only when fibrosis or steatosis replace the major part of the liver parenchyma. Interestingly, the normal sample of Patient 29 of the test set was partly misclassified due to severe steatosis and fibrosis; still, the normal tissue found at the border of the tumor sample, which is affected by moderate steatosis, was correctly classified, as shown in Fig. 6b.

Although fibrosis and steatosis may limit classification performances in some cases, the possibility to visualize fibrotic or steatotic changes of liver tissue in a label-free manner might offer additional advantages to this technique. Optical biopsies should be exploited towards other applications. Intraoperative real-time information about grading and classification of fibrosis, cirrhosis or steatosis may be helpful for intraoperative surgical strategy (e.g., acceptable extent of liver resection depending on the quality of liver parenchyma).

The investigation of formalin-fixed bulk tissue samples was an attempt to advance the research in the direction of intraoperative analysis of unprocessed tissue avoiding preparation of sections. However, the effects of fixation on tissue autofluorescence were so large to prevent the classification based on the training set of cryosection. A classification of formalin-fixed bulk tissue images that qualitatively agrees with the HE staining could be attained by exploiting the CARS signal alone, which is known to be unaffected by formalin fixation from previous studies^65^. The fixation (required for logistic issues) represented the main limitation of this study, which should be overstepped with a future study on freshly excised and totally unprocessed tissue samples, with the multiphoton microscopy system moving away from the research labs to the OR.

Future intraoperative exploitation can be foreseen both as rapid “live” microscopic imaging of excised samples avoiding all tissue preparation, as well as in situ endoscopic technique. Indeed, compact and portable multiphoton microscopes fitting the operation theater are not only available since years^66^, but are also certified medical products^67^. By contrast, endoscopes have to be highly efficient and enable video-rate imaging to comply with motion artefacts from surgeon handling as well as patient respiration and heartbeat. Although the technical feasibility of such systems was already demonstrated since a decade^68^, none has come yet on the market of medical instruments. Finally, yet importantly, all multiphoton systems (both microscopes and endoscopes) need to work with strongly reduced ambient light. This can be easily obtained with an enclosure integrated into a portable microscope. For all these reasons, a solution based on analysis of freshly resected tissue performed near or inside the OR seems to be closer to the medical practice.

In conclusion, label-free multiphoton microscopy has the potential for improving margins in metastatic liver surgery and should be further investigated toward translation into the surgical practice. Future studies should focus on unprocessed fresh tissue samples to demonstrate prognostic benefits for patients with liver metastases. Furthermore, investigations of primary hepatic cancers such as hepatocellular carcinoma may likewise help to increase the precision of resection, in the hypothesis that a sound differentiation between the neoplastic tissue and the strongly altered liver parenchyma where the carcinoma develop is possible.

## Materials and methods

### Study design: patients’ characteristics and tissue samples

Tissue samples from the tumor tissue bank of the University Cancer Center (UCC) Dresden, as well as bulk tissue samples from prospectively included patients undergoing liver resections for CRLM, were used for analysis. Clinical information (pretreatment and patient characteristics) for all patients with stored tissue samples in the UCC Dresden tissue bank was recorded in the UCC tissue bank database. Clinical information of prospectively included patients was recorded in specific case report forms.

One hundred patients that received liver resection for CRLM at the Department of Visceral-, Thoracic and Vascular Surgery at the Technische Universität Dresden between 2013 and 2018 were randomly selected in the UCC database of cryo-conserved tissue. Cryosections with 10 µm thickness of matched samples of CRLM and normal liver tissue were obtained from the tissue bank of UCC Dresden. Viable cryosections deemed from MPM imaging were available from 76 patients (matched normal and tumor tissue samples of 61 patients, only tumor tissue samples of 9 patients and only normal tissue samples of 6 patients). Samples were stored at − 80 °C until microscopy imaging.

In addition, fresh tissue samples containing the tumor border were prospectively collected from patients undergoing liver resection for CRLM at the Department of Visceral-, Thoracic and Vascular Surgery at the Technische Universität Dresden in 2020 and 2021. Inclusion criteria were informed consent, an age > 18 years and planned hepatectomy for CRLM (patients screened n = 76, included n = 70, liver tissue samples used for MPM imaging n = 30). Bulk tissue samples were stored in 4% formalin until MPM imaging.

The study cohort of samples obtained from the UCC tumor tissue bank comprised n = 76 patients. Most patients were males (n = 56), the median age was 64.5 years (min: 37, max: 81), and the median BMI was 26.1 kg/m^2^ (min: 17.7, max: 42.8). Patients that received neoadjuvant or conversion therapy in the two months preceding hepatectomy were n = 32 (chemotherapy only n = 11; chemotherapy plus monoclonal antibody n = 21). Patients’ information is given in Supplementary Table S1.

### Ethics statement

All tissue samples supplied by the Tumor and Normal Tissue Bank of the UCC Dresden were used in accordance with the rules of the tissue bank and with the vote of the local ethics committee of the Technische Universität Dresden (BO-EK-26012020). All fresh tissue samples were obtained from prospectively included patients that gave informed consent. The study was conducted according to the Declaration of Helsinki and approved by the local ethics committee of the Technische Universität Dresden (BO-EK-26012020). The study was registered by the German Clinical Trials Register (Trial ID: DRKS 00022261).

### Label-free MPM imaging

A laser scanning microscope (Axio Examiner Z.1 with scanning module LSM 7, Carl Zeiss AG, Jena, Germany) was used coupled with two picosecond lasers at 781 nm and 1005 nm (FemtoFiber pro NIR and FemtoFiber pro TNIR, respectively; both from Toptica Photonics AG, Gräfelfing, Germany). A 20× microscope objective with a numerical aperture of 1.0 was used for focalization, leading to a laser spot with a diameter of about 0.5 µm.

CARS and SHG were acquired in transmission on cryosections and in reflection on bulk samples, using pass-band emission filters with bands 626–654 nm and 380–400 nm, respectively. TPEF was always acquired in reflection using a bandpass filter of 500–550 nm. The dimension of the field of view (FoV) was 152 µm × 302 µm and it was scanned with 152 × 302 pixels, resulting in a pixel size of 1 µm. The pixel dwell was set to 3.4 µs, resulting in an acquisition time of 362 ms. A tiling procedure was used to acquire multiple FoVs covering a large sample area. At least 120 single FoV images with three channels (CARS, TPEF and SHG) were acquired from each sample, the actual number depending on cryosection dimensions. For visualization purposes, the three signal channels were encoded in an RGB image (red channel: CARS, green channel: TPEF, blue channel: SHG).

Cryosections were rehydrated in PBS and a coverslip was applied prior to MPM. Formal-fixed bulk biopsies were washed in PBS and embedded in low melting point agarose (LE Agarose, Biozym Scientific GmbH, Hessisch Oldendorf, Germany). A flat tissue surface for observation with MPM was produced by cutting with a vibratome (VT1200S, Leica Biosystems Nussloch GmbH, Nussloch, Germany). Prior to MPM, the sample was removed from PBS and a coverslip was applied on the observation surface. Acquisition of multiphoton images was done using the same parameters given above.

Additionally, selected regions of all samples (cryosections and bulk biopsies) were imaged with higher resolution (pixel dimension = 0.2 µm) and a twofold pixel averaging in order to obtain high-quality pictures for a qualitative evaluation of the subcellular structures.

In order to reduce background in the CARS channel and improve visibility of tissue structures in RGB images, the contrast was adapted in GIMP—GNU Image Manipulation Program 2.10. A linear correction of the signal intensity was applied to each individual channel of the whole image.

### Reference histology

All analyzed cryosections were stained with hematoxylin and eosin (HE). HE staining was performed directly on the same unfixed cryosections that were previously rehydrated and used for MPM imaging. Therefore, the overall quality is inferior compared to HE staining of paraffin-embedded tissue sections, which are the gold standard for histopathology. The color tones resulted here more brownish than usual, however cell nuclei and tissue structures were visible and could be interpreted by the pathologist.

Formalin-fixed bulk samples were embedded in cryomedium (Leica Biosystems Nussloch GmbH, Germany) and snap-frozen. Afterwards, cryosections with a thickness of 10 µm were prepared and the HE staining was performed.

### Texture analysis and automated image classification

Texture analysis and automated classification of each FoV image were performed using MATLAB R2021b (The MathWorks Inc., Natick, MA, USA) and all codes are provided in the Supplementary Information.

Each image channel was analyzed separately. After min–max normalization of each channel, 17 texture parameters were calculated from each channel using built-in MATLAB functions. The first-order parameters included mean gray value, standard deviation, kurtosis, skewness and entropy. Second-order parameters were also determined. The gray-level co-occurrence matrices were calculated in four orientations (0°, 45°, 90°, and 135°) and used to calculate contrast, correlation, energy, and homogeneity for three different distances (1, 12 µm and 30 µm). For each distance, texture parameters were obtained from the average of values calculated for the four orientations.

The texture parameters were used for the classification of tumor vs. liver tissue by means of linear discriminant analysis (MATLAB built-in function “classify”). The dataset was split into a training set for the development of the classification model and an independent test set for model evaluation. All images of each patient were used in either training or test set. The classification function provides a posterior probability of class assignment. Assignment to the class “tumor” or “normal” was performed if the corresponding posterior probability exceeded 0.5.

## Supplementary Information

Supplementary Information.

## Data Availability

The datasets generated during and/or analyzed during the current study are available from the corresponding author on reasonable request.
