# Supplementary material for: Label-free multiphoton microscopy enables histopathological assessment of colorectal liver metastases and supports automated classification of neoplastic tissue
Source: Sci Rep. 2023 Mar 15;13:4274. doi: 10.1038/s41598-023-31401-5 (PMC10017791; doi:10.1038/s41598-023-31401-5)
Supplement: Supplementary file 1 — Supplementary Information. [file 41598_2023_31401_MOESM1_ESM.pdf]

SUPPLEMENTARY INFORMATION

Label-free multiphoton microscopy enables histopathological assessment of colorectal liver metastases and supports automated classification of neoplastic tissue

Roberta Galli [1,*], Tiziana Siciliano [2], Daniela Aust [3,4], Sandra Korn [5], Katrin Kirsche [6], Gustavo B. Baretton [3,4], Jürgen Weitz [4,5], Edmund Koch [7], Carina Riediger [4,5]

[1] *Department of Medical Physics and Biomedical Engineering, Faculty of Medicine Carl Gustav Carus, Technische Universität Dresden, Fetscherstr. 74, D-01307 Dresden, Germany*

[2] *Center for Regenerative Therapies (CRTD), Technische Universität Dresden, Fetscherstr. 105, D-01307 Dresden, Germany.*

[3] *Institute of Pathology, University Hospital Carl Gustav Carus, Medical Faculty, Technische Universität Dresden, D-01307 Dresden, Germany*

[4] *National Center for Tumor Diseases (NCT/UCC), Partner Site Dresden: German Cancer Research Center (DKFZ), Im Neuenheimer Feld 280, D-69120 Heidelberg, Germany.*

[5] *Department of Visceral, Thoracic and Vascular Surgery, University Hospital Carl Gustav Carus, Technische Universität Dresden, D-01307 Dresden, Germany*

[6] *Neurosurgery, University Hospital Carl Gustav Carus, Technische Universität Dresden, Fetscherstr. 74, D-01307 Dresden, Germany*

[7] *Clinical Sensoring and Monitoring, Department of Anesthesiology and Intensive Care Medicine, Faculty of Medicine Carl Gustav Carus, Technische Universität Dresden, Fetscherstrasse 74, D-01307 Dresden, Germany*

** corresponding author: roberta.galli@tu-dresden.de*

Supplementary Table S1: Patient characteristics and classification results of normal tissue vs. tumor of FoV images in the test set and in the training set.

TEST SET									
						Normal		Tumor	
Pat. n.	Age @ OP	Gender	BMI (kg/m^2)	Chemotherapy	Antibody	N. of FoV	% correct	N. of FoV	% correct
1	60	M	31.8			122	0,95	338	1,00
2	75	F	22.7			101	0,99	208	1,00
3	71	M	38.4			83	0,99	333	1,00
4	63	F	30.1			123	0,99	309	0,95
5	38	M	25.7	x	x	239	1,00	114	0,79
6	65	M	34.0			224	0,99	362	1,00
7	56	M	32			139	0,88	255	1,00
8	49	M	26.8			351	1,00	558	0,91
9	61	F	26.3	x	x	177	1,00	113	1,00
10	60	M	26.1			267	0,97	331	0,95
11	62	M	22.1			186	1,00	155	0,77
12	58	M	25.2	x	x	240	1,00	236	0,98
13	68	M	23.9	x		412	1,00	367	1,00
14	70	M	29.4			190	0,67	385	1,00
15	71	M	30.9	x		276	1,00	152	0,84
16	65	F	26.0	x		421	1,00	40	0,05
17	63	M	27.1	x	x	281	1,00	447	0,71
18	58	M	20.3			252	1,00	147	0,69
19	62	M	31.2			251	0,96	396	0,73
20	72	M	22.9			527	0,99	696	0,96
21	56	F	21.8			355	1,00	265	0,98
22	65	F	42.8	x		222	1,00	344	0,99
23	46	M	19.6	x	x	272	0,99	209	0,93
24	68	M	28.4			204	1,00	253	1,00
25	67	F	22.4	x		154	0,97	311	0,99
26	52	M	24.1			183	0,79	210	0,95
27	57	M	28.7			463	0,82	370	0,96
28	65	M	26.8			212	0,97	195	1,00
29	51	M	18.3	x		386	0,72	480	0,69
30	62	M	22.8			401	0,98	301	0,86
31	56	F	26.2	x		415	0,94	125	0,88
32	44	F	26.2	x	x	700	0,98	734	0,97

TRAINING SET									
						Normal		Tumor	
Patinet n.	Age @ OP	Gender	BMI (kg/m^2)	Chemotherapy	Antibody	N. of FoV	% correct	N. of FoV	% correct
33	60	M	23.3	x		-		204	0.99
34	61	M	26.7			350	0.97	406	1.00
35	76	F	26.1			110	0.99	194	1.00
36	76	M	22.0			174	0.75	345	0.99
37	69	M	27.8	x	x	258	1.00	421	0.86
38	68	M	24.1			217	1.00	884	0.97
39	67	M	30.1			-		347	0.97
40	78	M	24.0			-		302	1.00
41	76	M	29.1			107	0.92	152	0.96
42	71	F	19.6			365	0.99	314	0.99
43	75	M	27.0			-		358	1.00
44	71	M	27.4			-		224	1.00
45	56	M	24.6	x	x	-		158	1.00
46	72	M	30.0			152	1.00	-	
47	49	M	24.2	x	x	89	0.99	-	
48	77	F	26.0			275	1.00	345	0.99
49	64	M	22.5	x	x	263	0.92	102	0.99
50	71	F	26.6	x	x	235	0.99	206	0.20
51	80	F	na	x		418	1.00	395	1.00
52	73	M	28.3			232	0.99	116	1.00
53	50	F	25.2	x	x	240	0.99	-	
54	46	M	22.9	x	x	489	1.00	107	0.95
55	74	F	17.8			372	0.99	-	
56	55	F	29.1			193	0.98	223	1.00
57	68	M	23.7			366	0.98	444	0.98
58	37	M	27.5	x	x	334	0.99	295	0.87
59	62	M	24.7	x		195	1.00	445	0.82
60	67	F	31.6	x		206	1.00	-	
61	65	M	26.8	x	x	-		148	0.56
62	67	F	21.2			337	1.00	351	1.00
63	72	M	20.3			323	0,88	507	1,00
64	46	M	19.6	x	x	269	0,98	224	0,99
65	46	M	19.1	x	x	245	1,00	-	
66	81	M	28.7	x	x	267	0,98	88	0,98
67	80	M	24.4	x	x	307	1,00	484	0,86
68	75	M	33.5			-		404	0,99
69	62	M	25.3			291	1,00	453	0,91
70	41	F	17.7	x	x	318	0,99	434	1,00
71	65	M	27.2			454	1,00	86	1,00
72	63	M	27.0	x	x	-		291	1,00
73	76	M	29.1			364	1,00	254	0,98
74	54	M	28.3			301	1,00	442	0,86
75	57	M	24.0			380	0,98	517	0,66
76	56	M	22.7			144	1,00	214	0,99

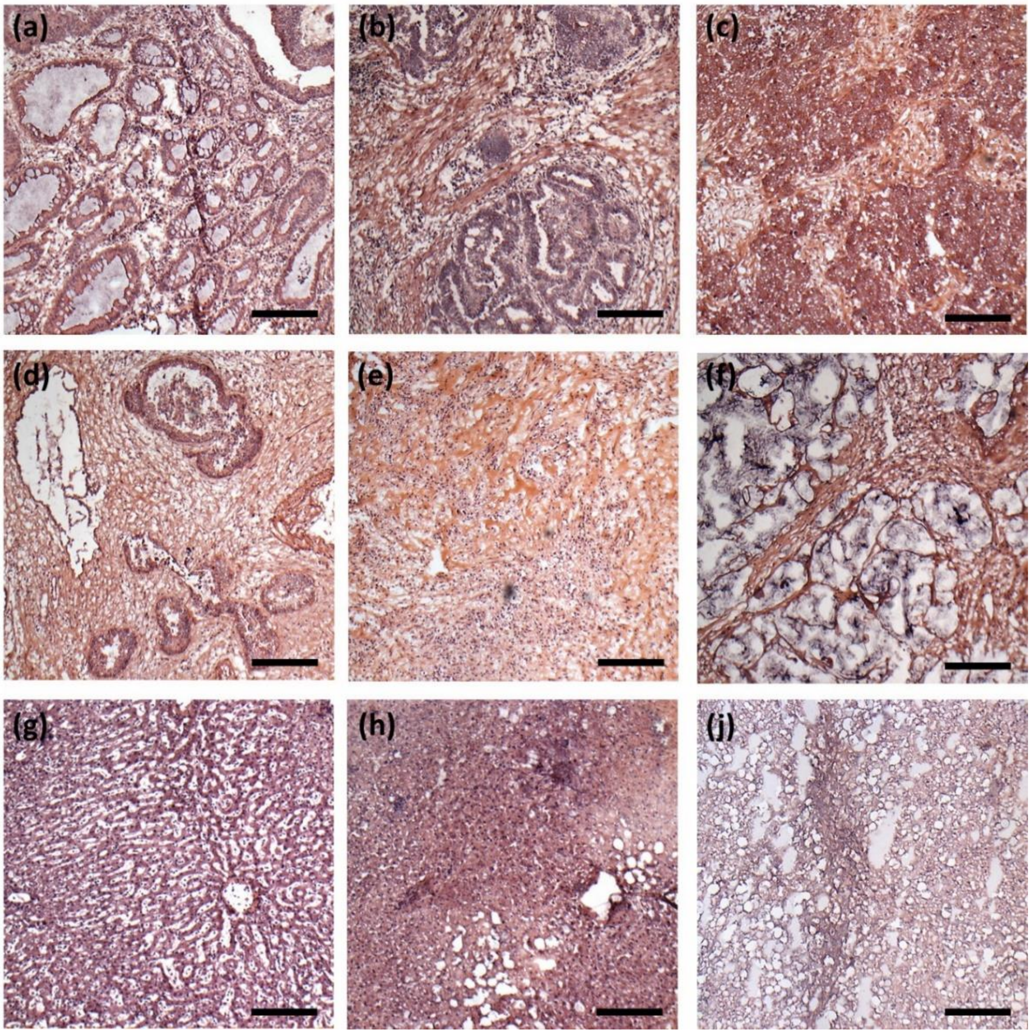

Supplementary Figure S1: HE staining images matching MPM images shown in Figure 1. Scale bar: 200 µm. (a)…(f): metastatic tissue; (g)…(j): non-metastatic liver tissue

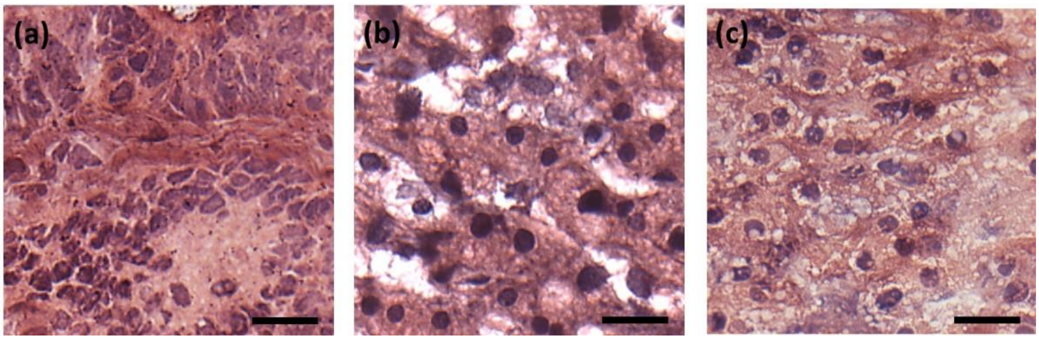

Supplementary Figure S2: HE staining images matching MPM images shown in Figure 2.
(a): metastatic tissue; (b), (c): non-metastatic liver tissue. Scale bar: 20 µm.

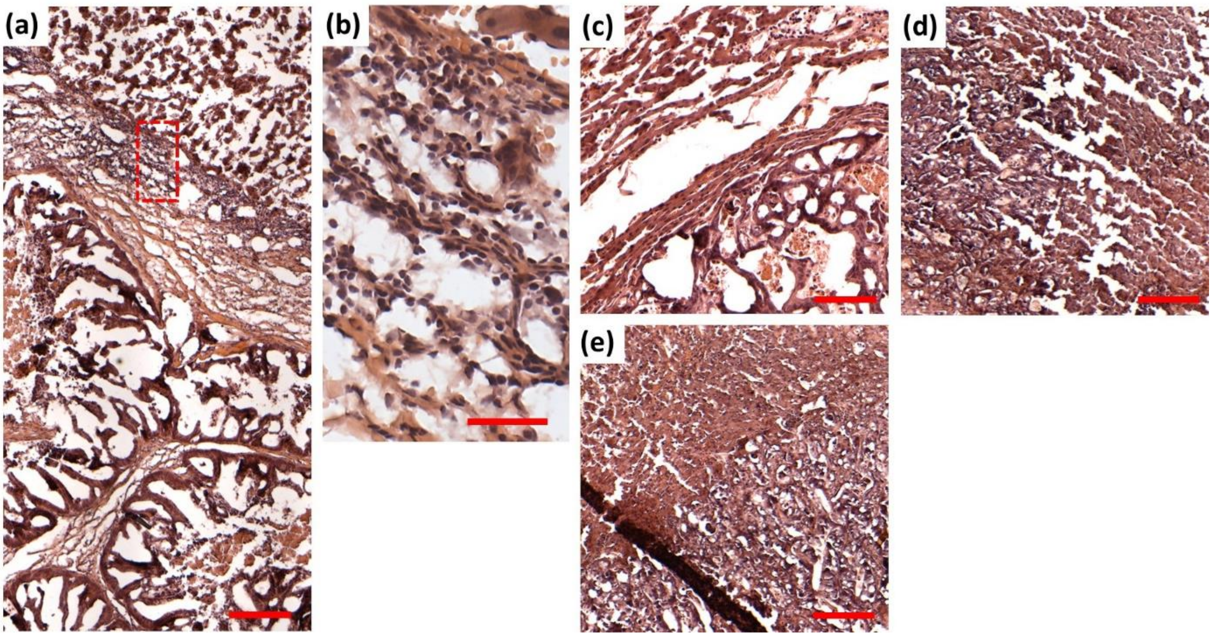

Supplementary Figure S3: HE staining images of samples shown in Figure 4. (a) desmoplastic HGP; (b) magnification of the border region in the box in (a); (c) pushing HGP; (d) replacement HGP; (e) Intermediate HGP between pushing and replacing. Scale bar in (a), (b)-(e): 200 µm; scale bar in (b): 50 µm.

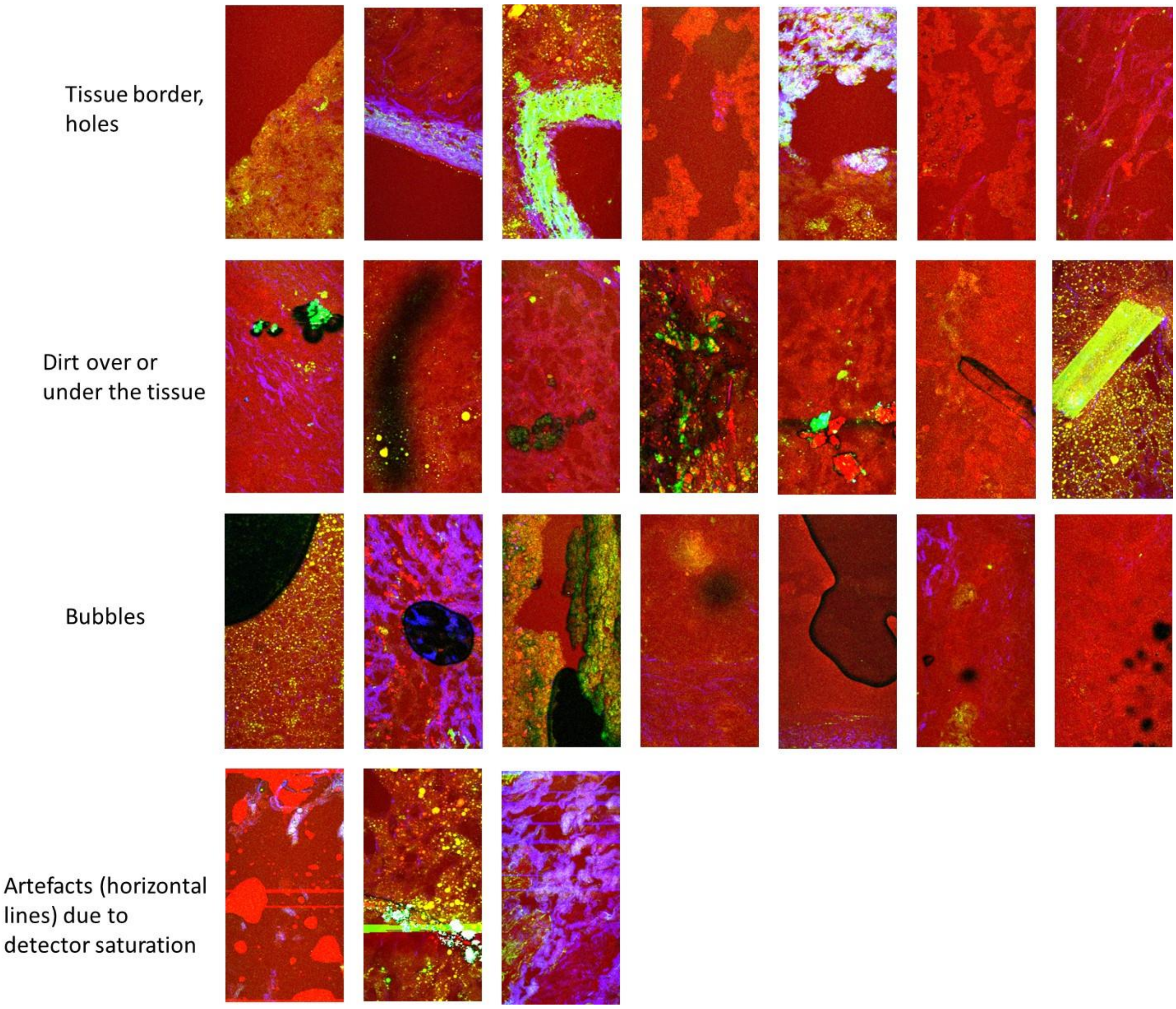

Supplementary Figure S4: Examples of MPM images excluded from automated classification because of different types of artefact.

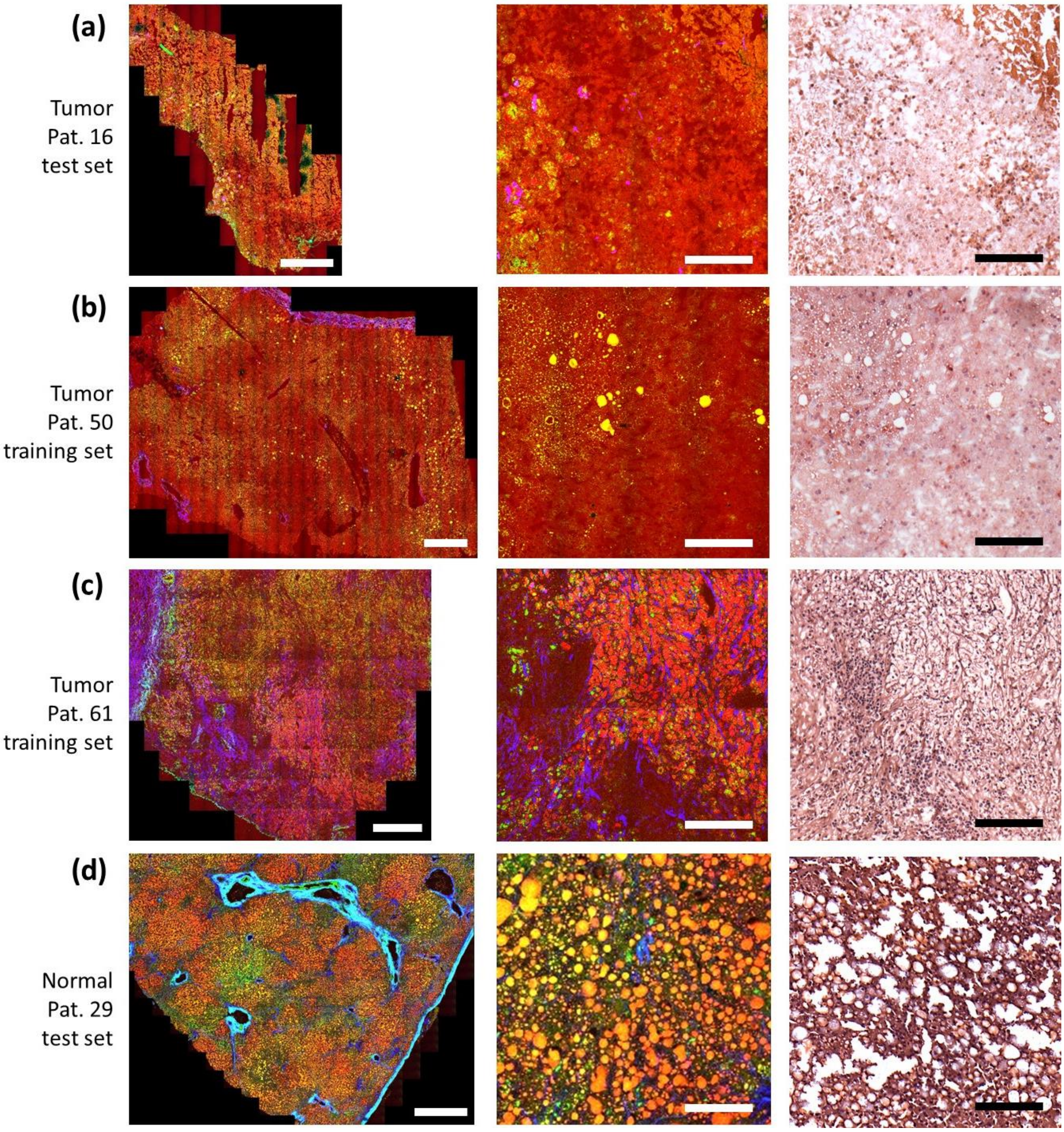

Supplementary Figure S5: Examples of misclassified samples. For each sample, an MPM image of the whole sample is shown on the left (scale bar: 500 µm), a detail of the MPM image roughly in the center (scale bar: 150 µm), and the matched HE staining on the right (scale bar: 150 µm). (a): Tumor sample of Patient 16. (b): Tumor sample of Patient 50. (c): Tumor sample of Patient 61. (d): Normal sample of Patient 29 (HE staining of a non-consecutive section).

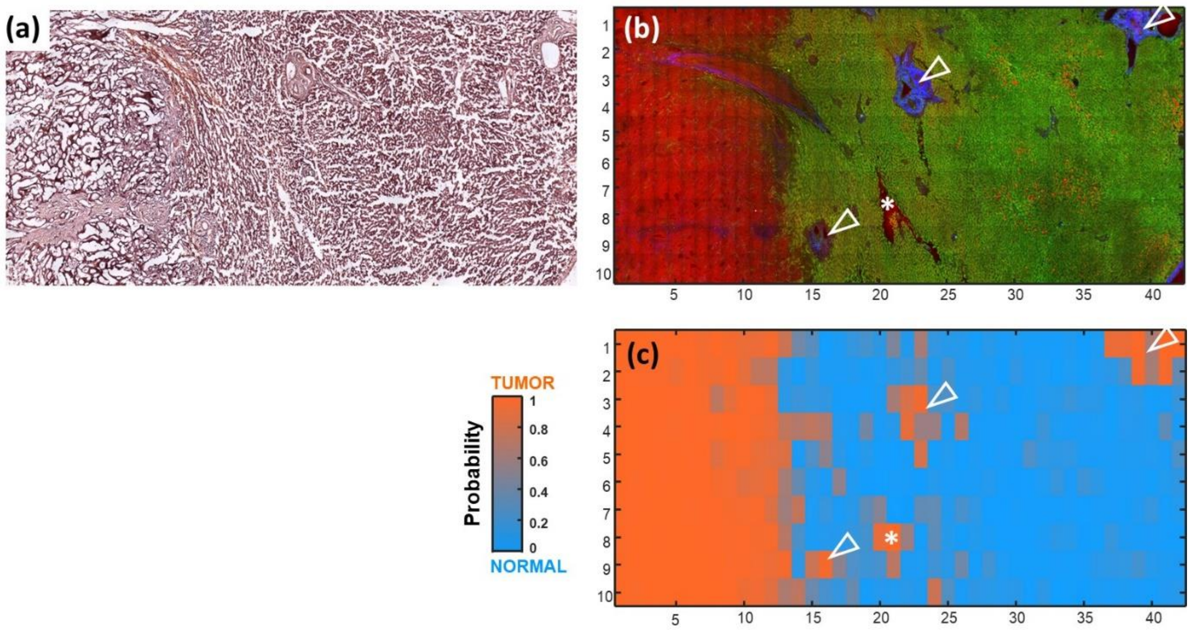

Supplementary Figure S6: Classification of formalin-fixed sample. (a): HE staining. (b): MPM tiled image. (c): map of posterior probability from the classification based on CARS texture parameters only. The posterior probability of classification of each single FoV image was color-coded as follows: 100% probability tumor: orange; 50% probability tumor: gray; 0% probability tumor: light blue. Axes' scale indicates the number of images. The border between tumor and normal liver tissue was correctly identified by image classification. Within the normal tissue region, classification errors (i.e. images classified as tumor) are related to presence of large blood vessels (arrowheads) or holes in the tissue (asterisk).

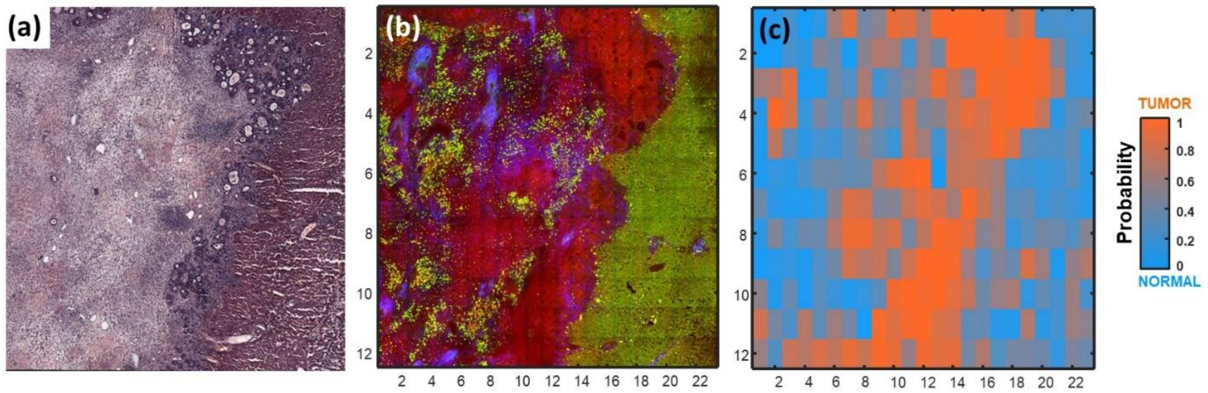

Supplementary Figure S7: Classification of formalin-fixed sample. (a): HE staining. (b): MPM tiled image. (c): map of posterior probability from the classification based on CARS texture parameters only. The posterior probability of classification of each single FoV image was color-coded as follows: 100% probability tumor: orange; 50% probability tumor: gray; 0% probability tumor: light blue. Axes' scale indicates the number of images. The regions of vital tumor and of normal liver tissue were correctly identified by image classification. A large necrotic region on the left side of the sample was classified as normal tissue.

Matlab codes

```
%load all images in a folder and create a matrix with all texture parameters
%in columns. Perform a min/max contrast enhancement.

sdirectory = uigetdir;
cd (sdirectory)     %select the directory where the txtfiles are.

tifffiles = dir([sdirectory '/*.tif']);

tic

texture_cars(1:17,1:length(tifffiles))=0;   % preallocation
texture_tpef(1:17,1:length(tifffiles))=0;   % preallocation
texture_shg(1:17,1:length(tifffiles))=0;   % preallocation


for k = 1:length(tifffiles)
    cd (sdirectory)
    filename = [sdirectory '/' tifffiles(k).name];
    A = imread(filename);
    A_cars=A(:,:,1);      %splitting the channels
    A_tpef=A(:,:,2);
    A_shg=A(:,:,3);

    A_cars=imadjust(A_cars,stretchlim(A_cars),[]);      %enhancing  contrast
min/max 1% saturation
    A_tpef=imadjust(A_tpef,stretchlim(A_tpef),[]);
    A_shg=imadjust(A_shg,stretchlim(A_shg),[]);


    cd ('C:/Roberta/work/texture')
    texture_cars(:,k) = texture_all_parameters(A_cars);
    texture_tpef(:,k) = texture_all_parameters(A_tpef);
    texture_shg(:,k) = texture_all_parameters(A_shg);
end

texture_cars=texture_cars';
texture_tpef=texture_tpef';
texture_shg=texture_shg';


clear A filename A_cars A_tpef A_shg
toc

function all_param = texture_all_parameters(A);


%FIRST ORDER

[m n]=size(A);
B=single(reshape(A,1,m*n));

%mean
meangray=mean(B);

%standard deviation
SDgray=std(B);

%kurtosis
kurt=kurtosis(B)-3;
```

```
%skewness
skew=skewness(B);

%entropy
entrop=entropy(A);

%SECOND ORDER
% gray-level co-occurrence matrices are calculated for 4 orientation (0°,
% 45°, 90° and 135°) and for two different offsets (given by D1 and D2).

D1=1;   % range on "near field" texture (1 pixel = 1 um)

glcm_near_0= graycomatrix(A,'Offset',[0 D1]);
glcm_near_45= graycomatrix(A,'Offset',[-D1 D1]);
glcm_near_90= graycomatrix(A,'Offset',[-D1 0]);
glcm_near_135= graycomatrix(A,'Offset',[-D1 -D1]);

D2=12;   % range on "mid field" texture

glcm_mid_0= graycomatrix(A,'Offset',[0 D2]);
glcm_mid_45= graycomatrix(A,'Offset',[-D2 D2]);
glcm_mid_90= graycomatrix(A,'Offset',[-D2 0]);
glcm_mid_135= graycomatrix(A,'Offset',[-D2 -D2]);

D3=30;   % range on "far field" texture

glcm_far_0= graycomatrix(A,'Offset',[0 D3]);
glcm_far_45= graycomatrix(A,'Offset',[-D3 D3]);
glcm_far_90= graycomatrix(A,'Offset',[-D3 0]);
glcm_far_135= graycomatrix(A,'Offset',[-D3 -D3]);

stats_near_0 = graycoprops(glcm_near_0,'Contrast Correlation Energy
Homogeneity');
stats_near_45 = graycoprops(glcm_near_45,'Contrast Correlation Energy
Homogeneity');
stats_near_90 = graycoprops(glcm_near_90,'Contrast Correlation Energy
Homogeneity');
stats_near_135 = graycoprops(glcm_near_135,'Contrast Correlation Energy
Homogeneity');

stats_mid_0 = graycoprops(glcm_mid_0,'Contrast Correlation Energy
Homogeneity');
stats_mid_45 = graycoprops(glcm_mid_45,'Contrast Correlation Energy
Homogeneity');
stats_mid_90 = graycoprops(glcm_mid_90,'Contrast Correlation Energy
Homogeneity');
stats_mid_135 = graycoprops(glcm_mid_135,'Contrast Correlation Energy
Homogeneity');

stats_far_0 = graycoprops(glcm_far_0,'Contrast Correlation Energy
Homogeneity');
stats_far_45 = graycoprops(glcm_far_45,'Contrast Correlation Energy
Homogeneity');
stats_far_90 = graycoprops(glcm_far_90,'Contrast Correlation Energy
Homogeneity');
stats_far_135 = graycoprops(glcm_far_135,'Contrast Correlation Energy
Homogeneity');

% the texture parameters with offset D1, D2 and D3 are calculated as averages
% on the four directions.
```

```
contr_near=(stats_near_0.Contrast+stats_near_45.Contrast+stats_near_90.Cont
rast+stats_near_135.Contrast)/4;
contr_mid=(stats_mid_0.Contrast+stats_mid_45.Contrast+stats_mid_90.Contrast
+stats_mid_135.Contrast)/4;
contr_far=(stats_far_0.Contrast+stats_far_45.Contrast+stats_far_90.Contrast
+stats_far_135.Contrast)/4;

corr_near=(stats_near_0.Correlation+stats_near_45.Correlation+stats_near_90
.Correlation+stats_near_135.Correlation)/4;
corr_mid=(stats_mid_0.Correlation+stats_mid_45.Correlation+stats_mid_90.Cor
relation+stats_mid_135.Correlation)/4;
corr_far=(stats_far_0.Correlation+stats_far_45.Correlation+stats_far_90.Cor
relation+stats_far_135.Correlation)/4;

energy_near=(stats_near_0.Energy+stats_near_45.Energy+stats_near_90.Energy+
stats_near_135.Energy)/4;
energy_mid=(stats_mid_0.Energy+stats_mid_45.Energy+stats_mid_90.Energy+stat
s_mid_135.Energy)/4;
energy_far=(stats_far_0.Energy+stats_far_45.Energy+stats_far_90.Energy+stat
s_far_135.Energy)/4;

homog_near=(stats_near_0.Homogeneity+stats_near_45.Homogeneity+stats_near_9
0.Homogeneity+stats_near_135.Homogeneity)/4;
homog_mid=(stats_mid_0.Homogeneity+stats_mid_45.Homogeneity+stats_mid_90.Ho
mogeneity+stats_mid_135.Homogeneity)/4;
homog_far=(stats_far_0.Homogeneity+stats_far_45.Homogeneity+stats_far_90.Ho
mogeneity+stats_far_135.Homogeneity)/4;

all_param = ([meangray SDgray kurt skew entrop contr_near contr_mid contr_far
corr_near corr_mid corr_far energy_near energy_mid energy_far homog_near
homog_mid homog_far])';
```
